# The advanced glycation end-products (AGEs)/ROS/NLRP3 inflammasome axis contributes to delayed diabetic corneal wound healing and nerve regeneration

**DOI:** 10.7150/ijbs.63219

**Published:** 2022-01-01

**Authors:** Luqin Wan, Xiaofei Bai, Qingjun Zhou, Chen Chen, Huifeng Wang, Ting Liu, Junfa Xue, Chao Wei, Lixin Xie

**Affiliations:** 1 Qingdao Eye Hospital of Shandong First Medical University, Qingdao, 266071, China.; 2 State Key Laboratory Cultivation Base, Shandong Provincial Key Laboratory of Ophthalmology, Shandong Eye Institute, Shandong First Medical University & Shandong Academy of Medical Sciences, Qingdao 266071, China.

**Keywords:** diabetic keratopathy, NLRP3 inflammasome, advanced glycation end products, corneal wound healing, reactive oxygen species

## Abstract

Diabetic keratopathy (DK) is an important diabetic complication at the ocular surface. Chronic low-grade inflammation mediated by the NLRP3 inflammasome promotes pathogenesis of diabetes and its complications. However, the effect of the NLRP3 inflammasome on DK pathogenesis remains elusive. Wild-type (WT) and* Nlrp3* knockout (KO) C57BL/6 mice were used to establish a type I diabetes model by intraperitoneal injection of streptozotocin. The effect of the NLRP3 inflammasome on diabetic corneal wound healing and never regeneration was examined by a corneal epithelial abrasion model. Western blot, immunofluorescence staining, enzyme-linked immunosorbent assay (ELISA) and pharmacological treatment were performed to investigate the regulatory mechanism of advanced glycation end products (AGEs) on NLRP3 inflammasome activation and corneal wound healing *in vivo*. The cultured mouse corneal epithelial cells (TKE2) were used to evaluate the effect and mechanism of AGEs on NLRP3 inflammasome activation* in vitro*. We revealed that NLRP3 inflammasome-mediated inflammation and pyroptosis contributed to DK pathogenesis. Under physiological conditions, the NLRP3 inflammasome was required for corneal wound healing and nerve regeneration. However, under a diabetic scenario, sustained activation of the NLRP3 inflammasome resulted in postponed corneal wound healing and impaired nerve regeneration. Mechanistically, the accumulated AGEs promoted hyperactivation of the NLRP3 inflammasome through ROS production. Moreover, genetically and pharmacologically blocking the AGEs/ROS/NLRP3 inflammasome axis significantly expedited diabetic corneal epithelial wound closure and nerve regeneration. Our results revealed that AGEs-induced hyperactivation of the NLRP3 inflammasome resulted in delayed diabetic corneal wound healing and impaired nerve regeneration, which further highlighted the NLRP3 inflammasome as a promising target for DK treatment.

## Introduction

Diabetes mellitus (DM) characterized by long-term hyperglycaemia and various complications has become a major health issue worldwide, with nearly 463 million people suffering from diabetes in 2019 1. The ophthalmic complications due to diabetes have been reported as a leading cause affecting eye health, including diabetic retinopathy, diabetic cataract, and diabetic keratopathy (DK) 2,3. About 47%-64% of diabetic patients were estimated to suffer from DK 4, usually with pathological alterations, such as delayed corneal wound healing, reduced corneal subepithelial nerve density, and impaired corneal sensation 2. More seriously, uncontrolled postponed corneal wound healing probably increased the susceptibility to corneal ulcers, microbial keratitis, and even perforation 2,5. Therefore, a better understanding of the pathogenesis of DK is vital for treating and preventing this scenario. Although increasing evidence indicated that low-grade chronic inflammation contributes to the pathogenesis of diabetes and its complications 6-9, the associations of chronic inflammation with DK currently remain uncertain.

The inflammasomes are a cytosolic signalling complex, which usually contains a sensor NOD-like receptor (NLR), the adaptor protein ASC, and Caspase-1 (Casp-1) 10. As the best-characterized inflammasome, the NLRP3 inflammasome can be activated by a broad range of stimuli, including pathogenic molecules, sterile insults, and metabolic products through efflux of potassium ions, flux of calcium ions, lysosomal disruption, mitochondrial dysfunction, metabolic changes, and trans-Golgi disassembly 11-13. The assembly of the NLRP3 inflammasome results in caspase-1-dependent secretion of interleukin (IL)-1β and IL-18, as well as gasdermin D (GSDMD)-mediated pyroptosis 14,15. Accumulating evidence revealed that NLRP3-inflammasome-mediated chronic inflammation contributed to the development and progression of DM and its complications, such as diabetic nephropathy 6, diabetic retinopathy 16,17, diabetic cardiomyopathy 18, and diabetes-associated atherosclerosis 19. Moreover, the hyperactivation of the NLRP3 inflammasome was reported to be involved in impaired cutaneous wound healing in diabetic mice and humans 20,21. However, whether the NLRP3 inflammasome contributes to the pathogenesis of DK remains largely unknown.

In this study, we first examined the effect of NLRP3 inflammasome on corneal epithelial wound using streptozotocin (STZ)-induced diabetic mice and then focused on the pathogenic mechanism of the NLRP3 inflammasome involved in. Our results demonstrated that the persistent NLRP3 inflammasome activation contributed to delayed diabetic corneal wound healing and impaired nerve regeneration. Mechanistically, the accumulated AGEs promoted the hyperactivation of the NLRP3 inflammasome through reactive oxygen species (ROS), ultimately leading to increased pyroptosis and impaired proliferative capacity of corneal epithelial cells. These findings highlighted the pathogenic roles of the NLRP3 inflammasome in DK.

## Materials and methods

### Animals

Male C57BL/6 mice (6-8 weeks old) were purchased from the Vital River Laboratory Animal Technology Co., Ltd. (Beijing, China) and housed in the animal center of Shandong Eye Institute. All animal experiments were conducted with the approval of the Ethics Committee of Shandong Eye Institute and carried out following the Research in Vision and Ophthalmology Statement for the Use of Animals in Ophthalmic and Vision Research. According to our previous study 22, type 1 diabetes was induced in wild-type (WT) and *Nlrp3* knockout (KO) mice through intraperitoneal injection of STZ (50 mg/kg, Sigma-Aldrich, St. Louis, MO) for 5 consecutive days. The OneTouch Basic glucometer (Life Scan, Johnson & Johnson, Milpitas, CA) was employed to monitor the level of blood glucose. Diabetic mice were used for subsequent experiments at 16 weeks after the final STZ injection, with blood glucose values at 29.88 ± 3.36 mmol/L (Supplementary Fig. 1).

### Corneal epithelial wound healing and treatment

To build the corneal epithelial debridement model, normal and diabetic mice of the same age were anesthetized by 0.6% pentobarbital sodium, and the central corneal epithelium (2.75 mm diameter) in one eye of each mouse was removed using an Algerbrush II corneal rust ring remover (Alger Co., Lago Vista, TX). After 0, 24, and 48 hours, the residual epithelial defects in the cornea were visualized by 0.25% fluorescein sodium and photographed under a slit lamp microscope (BQ900; Haag-Streit, Bern, Switzerland). The percentage of the defect area was quantified with Image J software (version 1.47, National Institutes of Health, Bethesda, MD) as our previous descriptions 23.

To determine the effect of the NLRP3 inflammasome on corneal wound closure, MCC950 (5 μL, 100 μg/mL, MCE) was subconjunctivally administrated immediately after abrasion. To evaluate the effect of corneal wound healing on oxidative stress, diabetic mice were topically treated with ROS quencher N-acetylcysteine (NAC, 5μL, 2.5μg/μL, Sigma) at 0 hours after corneal epithelial abrasion. Moreover, to examine the pathological role of AGE on corneal wound healing, WT mice were subconjunctivally injected with AGE-BSA (5 μL, 10 μg/μL, Abcam) at 0 hours after the removal of corneal epithelium. Phosphate-buffered saline (PBS) was used as control. To further investigate the effect of AGEs on diabetic corneal wound closure, pyridoxylamine (PM, 1g/L, MCE, an AGE inhibitor 24) in drinking water was given to the diabetic mice for two months.

### Corneal whole-mount staining for nerve fibres

Corneal whole-mount immunofluorescence staining was performed, as in previous descriptions 25. Briefly, mouse eyeballs were immediately fixed in Zamboni stationary liquid (Solarbio) for 1 h after collection; the corneas were then dissected and blocked for 2 hours in PBS containing 0.1% Triton X-100, 2% goat serum, and 2% bovine serum albumin. Subsequently, the corneas were incubated overnight at 4 °C with Alexa Fluor 488 conjugated neuronal class III β-tubulin mouse monoclonal antibody (Merck-Millipore, Darmstadt, Germany). After washing each cornea six times, the corneas were cut into six petals and observed using a confocal microscope (Zeiss, Rossdorf, Germany). The density of the corneal sub-basal nerve fibres was quantified by Image J software.

### Corneal mechanical sensitivity measurement

Cochet-Bonnet esthesiometer (Luneau Ophtalmologie, Chartres Cedex, France) was used to measure corneal sensitivity in unanaesthetised normal and diabetic mice before and after scratching the epithelium, according to our previous protocol 23. The longest filament length with a positive response (which triggered the eyeblink response) was considered as the threshold of sensitivity. The test was repeated at least three times.

### Corneal epithelial cell culture and treatment

Mouse corneal epithelial cell line (TKE2) was provided by Dr. Tetsuya Kawakita of Keio University (Tokyo, Japan) 26. TKE2 cells were cultured in keratinocyte serum-free medium (Gibco, USA), containing human keratinocyte growth supplement (HKGS, 10 μL/mL) and epidermal growth factor (EGF, 5 ng/mL). After starvation overnight, the cultured cells were treated with bovine serum albumin (BSA) (200 μg/mL) and AGE-BSA (200 μg/mL, Abcam) for 24 hours. To inhibit the NLRP3 inflammasome, the cells were co-treated with AGE-BSA (200 μg/mL) and MCC950 (10 μM/mL, MCE) for 24 hours. The treated cells were then harvested for CCK-8 analysis, immunostaining and western blot, and the supernatants were prepared for the enzyme-linked immunosorbent assay (ELISA) test.

### Immunofluorescence staining

Immunofluorescence staining was performed according to our previous protocol 25. Mouse eyeballs were collected and embedded immediately in the OCT compound (Sakura Finetek, Tokyo, Japan) for frozen sections. The 7 μm-thick sections and treated TKE2 cells were fixed in 4% paraformaldehyde for 15 min, permeabilized with 0.1% Triton X-100, and blocked with 5% BSA for 1h at room temperature. The processed samples were incubated with primary antibodies (as shown in Supplementary Table 1) overnight at 4 °C and subsequently with fluorescein-conjugated secondary antibodies. All samples were photographed by an Eclipse TE2000-U microscope (Nikon, Tokyo, Japan) after counterstaining with DAPI.

### Western blot

The total proteins were extracted from mouse corneal tissues and TKE2 cells in radioimmunoprecipitation assay (RIPA) buffer containing a proteinase inhibitor cocktail. The extracted proteins were separated by 10% or 12.5% SDS-PAGE gels and then transferred to polyvinylidene fluoride membranes (Millipore, Billerica, MA). The 5% BSA was used to block the protein-loaded membranes for 1 hour at room temperature. After washing with TBST, the blots were incubated overnight at 4 °C with primary antibodies (as shown in Supplementary Table 1) and then with species-specific secondary antibodies for 1 hour at room temperature. Protein-specific signals were visualized using the Enhanced Super Signal Chemiluminescent Substrate (Thermo Fisher Scientific). The images were obtained using a chemidoc^TM^ touch imaging system (Bio-Rad, Hercules, CA, USA) and quantified by Image J software.

### Enzyme-linked immunosorbent assay (ELISA)

The corneal tissues of diabetic mice and age-matched vehicle mice were collected at 48 hours after corneal epithelial abrasion (2.75 mm diameter). Three corneas from each group were homogenized in cold PBS. The Bicinchoninic Acid (BCA) Kit (Beyotime, Shanghai, China) was used to determine the total protein concentrations, and the levels of interleukin-1β (IL-1β) were then quantified using a commercially available ELISA kit (mouse sensitive kits for IL-1β, Proteintech, USA) according to manufacturers' protocol with a multifunctional microplate reader (SpectraMax i3x, Molecular Devices, Sunnyvale, CA, USA). The content of IL-1β in the TKE2 cell supernatant was also measured by the same method.

### Reactive oxygen species (ROS) staining

To observe the intracellular ROS staining, fresh eyeballs were embedded in the OCT compound and prepared for frozen sections immediately. Corneal cryostat sections were washed and incubated with dichloro-dihydro-fluorescein diacetate (DCFH-DA, 10 μmol/L) for 30 min at 37 °C. After DAPI staining, the intensity of fluorescence was observed and captured using an Inverted fluorescence microscope (ECHO, USA). To further determine the intracellular ROS in cells, the treated TKE2 cells were washed and incubated with DCFH-DA (10 μmol/L) for 30 min at 37 °C, and then the intensity of fluorescence was captured.

### Immunohistochemistry (IHC)

The harvested corneas from different groups were fixed in 4% paraformaldehyde, dehydrated and embedded in paraffin. The deparaffinized sections (5 µm) were rehydrated and then antigen was retrieved using citrate antigen retrieval solution. After removing endogenous peroxidase, the sections were blocked with 5% BSA at room temperature. Antibody specific for AGE (showed in Supplementary Table 1) was incubated at 4 °C overnight. The sections were then incubated with appropriate secondary antibodies for 50 min at room temperature. Haematoxylin was used to counterstain the nuclei. Finally, photographs were taken under a light microscope after dehydration (Olympus, Japan).

### CCK-8 detection

The cell counting kit-8 (Bioss, Beijing, China) was used to detect cell proliferative ability. The TKE2 cells (1000 per well) were seeded into 96-well plates with the corresponding medium. The kit solution was added to the plate for 2 hours. The optical density (OD) value of each well was detected at the wavelength 450 nm using a Microplate reader (Model 680; Bio-199 Rad, Hercules, CA). Each assay was conducted at least in triplicate.

### Data analysis

Statistical analyses were performed using SPSS version 19.0 software (IBM Corporation, Chicago, IL). All data were presented as the means ± standard deviation (SD). Student's two-tailed t-test was used to compare two groups. One-way analysis of variance was used to analyse experiments with more than two groups. Differences were considered statistically significant at a P value of less than 0.05.

## Results

### NLRP3 inflammasome promotes normal corneal epithelial repair and nerve regeneration

**Figure 1:**
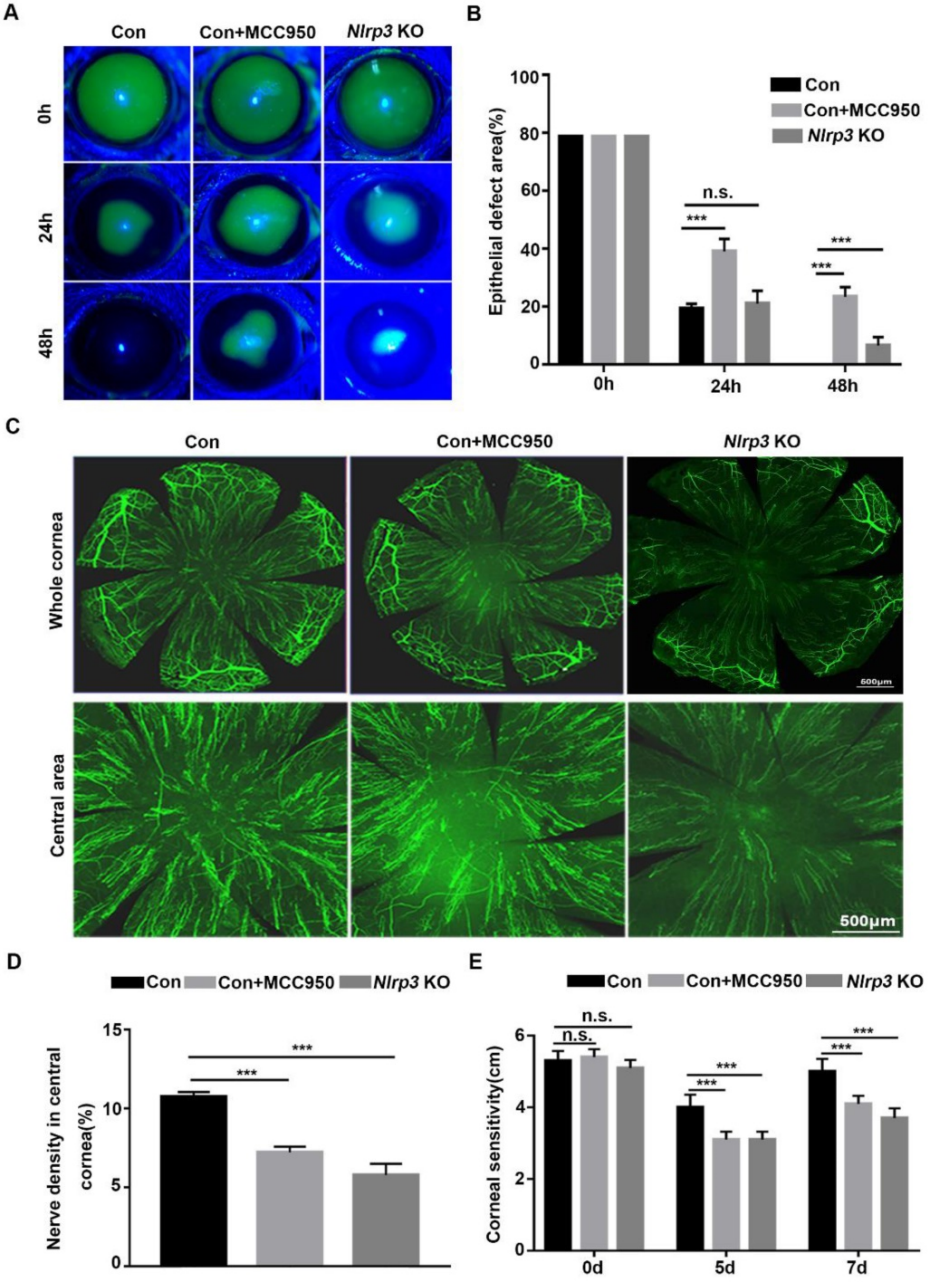
** NLRP3 inflammasome promoted normal corneal epithelial repair and nerve regeneration. (A)** The dynamic changes of corneal defects in three groups at 0 h, 24 h and 48 h after epithelial abrasion were observed by fluorescein sodium staining. **(B)** The wound sizes of corneas as in (A) were calculated by Image J software (n=5). **(C)** Seven days after epithelial abrasion, β-tubulin III staining was performed to determine the alterations of regenerated corneal nerve fibers. The representative images of the entire cornea were shown in the top panels, and the central cornea images were presented in the bottom panels. **(D)** The nerve densities of the central cornea on the basis of the areas staining positive for β-tubulin III were calculated by Image J software (n=5). **(E)** The corneal sensation was tested by a Cochet-Bonnet esthesiometer at 5 and 7 d after corneal epithelial abrasion. The results were descripted as mean ±SD. n.s, not significant, *** *P* <0.001.

To explore the effect of the NLRP3 inflammasome on corneal wound healing and nerve regeneration under physiological conditions, WT and *Nlrp3* KO mice were used to establish a corneal epithelial injury model. When compared with WT mice (Control), the *Nlrp3* KO mice showed significantly delayed corneal epithelial wound closure and lower density of nerve fibers in regenerated corneal epithelium, as well as reduced corneal sensitivity (Fig. 1A-E). These findings were consistent with the results obtained by inhibiting the NLRP3 inflammasome through MCC950 (as shown in the Control +MCC950 group), which also pronouncedly displayed postponed corneal wound healing and impaired nerve regeneration (Fig. 1A-E). Collectively, both genetic and pharmacologic evidence suggested that the NLRP3 inflammasome was required for corneal wound closure and nerve regeneration under normal physiological conditions.

### The NLRP3 inflammasome was hyperactivated during diabetic corneal wound healing

**Figure 2:**
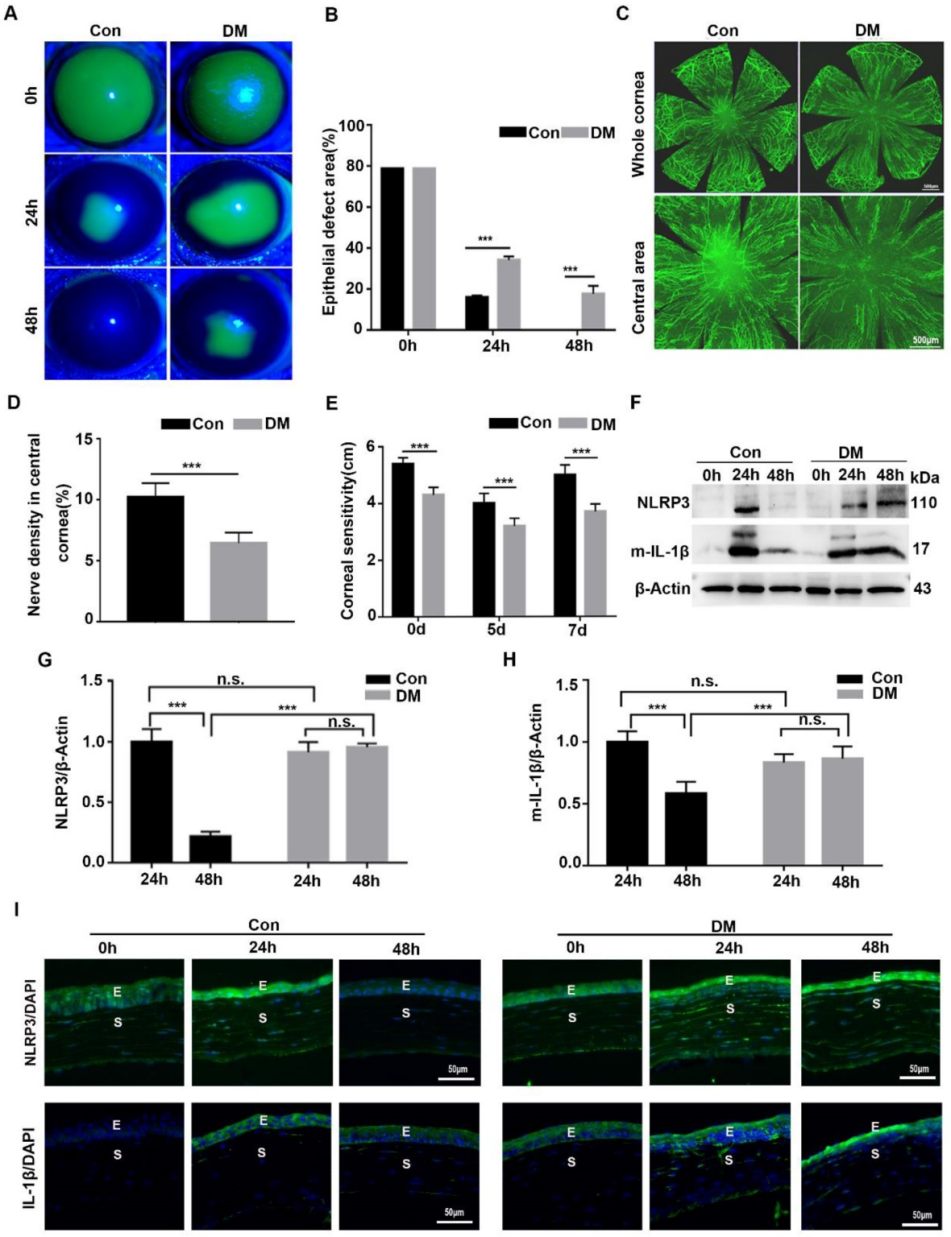
** NLRP3 inflammasome was hyperactivated during diabetic corneal wound healing. (A)** The representative images of corneal defects in two groups at 0h, 24 h and 48 h after epithelial abrasion were shown by fluorescein sodium staining. **(B)** The corneal defects area as in (A) were quantified by Image J (n=5). **(C)** The representative images of regenerated corneal nerve fibers were observed using β-tubulin III staining at 7d after epithelial abrasion. The top panel, the whole corneas; the bottom panel, the central corneas. **(D)** The nerve densities of central cornea between two groups (n=5) were determined by Image J software. **(E)** The corneal sensitivity recovery between two groups (n=5) was quantified using a Cochet-Bonnet esthesiometer at 5 and 7 d after corneal epithelial abrasion. **(F)** The levels of NLRP3 and matured IL-1β (m-IL-1β) in the corneas at different time points after corneal epithelial debridement were tested via western blot. **(G-H)** The relative levels of the NLRP3 and m-IL-1β expression were calculated by Image J software as in (F) (n=3). **(I)** The expression of NLRP3 and IL-1β during corneal epithelial regeneration was evaluated by immunofluorescence staining. Scale bar, 50 µm. E, epithelium; S, stroma. The results were expressed as mean ±SD. n.s, not significant, *** *P* <0.001.

Although we recognized the importance of NLRP3 inflammasome during normal corneal wound closure, the effect of the NLRP3 inflammasome on diabetic corneal wound healing remained uncertain. We first investigated the activation of the NLRP3 inflammasome during diabetic wound closure. As shown in Fig. 2A-B, the corneal wound closure in diabetic mice was more significantly postponed than that in the Control group, with their closure ability (65.9% VS 84.2%) on 24 hours after debridement, and (82.4% VS 100%) on 48 hours after debridement. The density of nerve fibers and the corneal sensitivity recovery in the DM group was much lower than that in the control group (Fig. 2C-E). These findings were in line with our previous studies. Furthermore, we found that the protein level of NLRP3 in the control corneas was pronouncedly elevated at 24 hours and lowered at 48 hours after debridement. A similar dynamic trend of matured IL-1β was also observed in Control corneas during wound healing. In contrast, the levels of NLRP3 and matured IL-1β in diabetic corneas were dramatically increased at 24 hours and sustained a high expression at 48 hours (Fig. 2F-H). Correspondingly, the immunofluorescence staining also showed sustained higher expression of NLRP3 and IL-1β in diabetic corneas than in control group during wound healing (Fig. 2I). Moreover, using immunostaining, we also found increased oxidative stress during diabetic corneal wound closure, characterized by aggravated ROS accumulation, and elevated expression of NADPH oxidase 2 (NOX2) and NOX4 in the corneal epithelial layer (Supplementary Fig. 2), which was reported to be related to NLRP3 inflammasome activation 27. Taken together, the findings indicated that the sustained activation of NLRP3 inflammasome probably contributed to the postponed diabetic corneal wound closure.

### Blocking NLRP3 inflammasome activation accelerated the diabetic corneal wound healing and nerve regeneration

**Figure 3:**
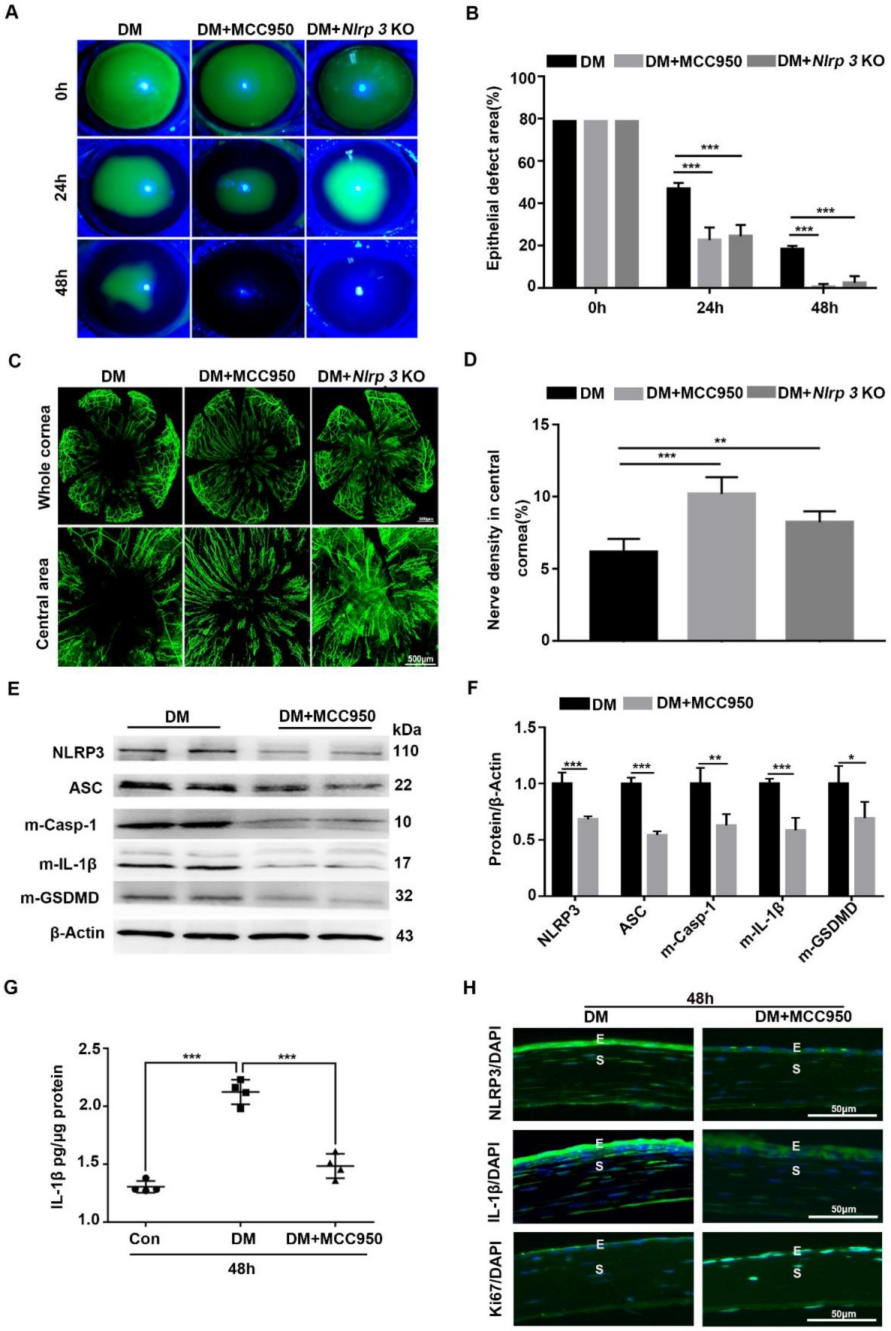
** Blocking NLRP3 inflammasome activation accelerated diabetic corneal epithelial wound healing and nerve regeneration. (A)** The typical photographs of corneal defects in three groups at 0 h, 24 h and 48 h after epithelial abrasion were taken using a slit-lamp microscope. **(B)** The areas of corneal epithelial defects within three groups at different time points (n=5) were quantified using Image J software. **(C)** The representative pictures of regenerated corneal nerve fibers in different groups (n=5) at 7d after epithelial abrasion were obtained using β-tubulin III staining. Upper panel, the whole corneas; bottom panel, the central corneas. **(D)** The nerve densities of central corneas in different groups (n=5) were quantified using Image J software. **(E)** The protein levels of NLRP3, ASC, matured Caspase-1 (m-Casp-1), m-IL-1β and matured GSDMD (m-GSDMD) in the corneas of different groups (n=3) at 48 h after corneal epithelial injury were determined by western blot. **(F)** The relative values of NLRP3, ASC, m-Casp-1, m-IL-1β and m-GSDMD in the corneas at 48 h after corneal epithelial injury were analyzed through Image J software as in (E). **(G)** The protein level of IL-1β in the corneas of different groups (n=4) at 48 h after epithelial abrasion were quantified by ELISA. **(H)** The expression of NLRP3, IL-1β and Ki67 in the corneas of different groups at 48 h after injury was examined using immunofluorescence staining. Scale bar, 50 µm. E, epithelium; S, stroma. Data were given as mean ±SD. *P <0.05, ** *P* <0.01, *** *P* <0.001.

Given the sustained activation of the NLRP3 inflammasome during diabetic wound healing, *Nlrp3* KO diabetic mice were established through intraperitoneal injection of STZ to evaluate its effect on corneal wound closure. When compared with diabetic WT mice, the *Nlrp3* KO diabetic mice displayed expedited corneal epithelial wound healing (Fig. 3A-B) and nerve regeneration (Fig. 3C-D), as well as a significant improvement in corneal sensation recovery (Supplementary Fig. 3A), suggesting the pathogenic role of the NLRP3 inflammasome in diabetic wound healing. Subsequently, we also evaluated the effect of the NLRP3 inflammasome on diabetic corneal wound healing using MCC950. The topical application of MCC950 significantly promoted diabetic corneal wound healing (Fig. 3A-B), nerve regeneration (Fig. 3C-D), and corneal sensation recovery (Supplementary Fig. 3A), which was consistent with the findings obtained from *Nlrp3* KO diabetic mice. In addition, MCC950 treatment increased Ki67 positive epithelial cells (Fig. 3H) and enhanced the expression of signal transducer and activator of transcription 3 (STAT3) (Supplementary Fig. 3B), which was associated with accelerated corneal epithelial wound healing 28. Furthermore, western blot, ELISA and immunofluorescence staining both revealed that the MCC950 treatment pronouncedly inhibited NLRP3 inflammation activation during diabetic wound healing, along with a reduction in matured Casp-1, IL-1β and GSDMD, as well as lower expression of NLRP3 (Fig. 3E-H). Overall, these results acquired through genetic and pharmacological approaches demonstrated that NLRP3-inflammasome-mediated inflammation led to delayed diabetic corneal wound healing and nerve regeneration.

### The diabetic corneas presented increased AGE deposition and basal activation of the NLRP3 inflammasome

**Figure 4:**
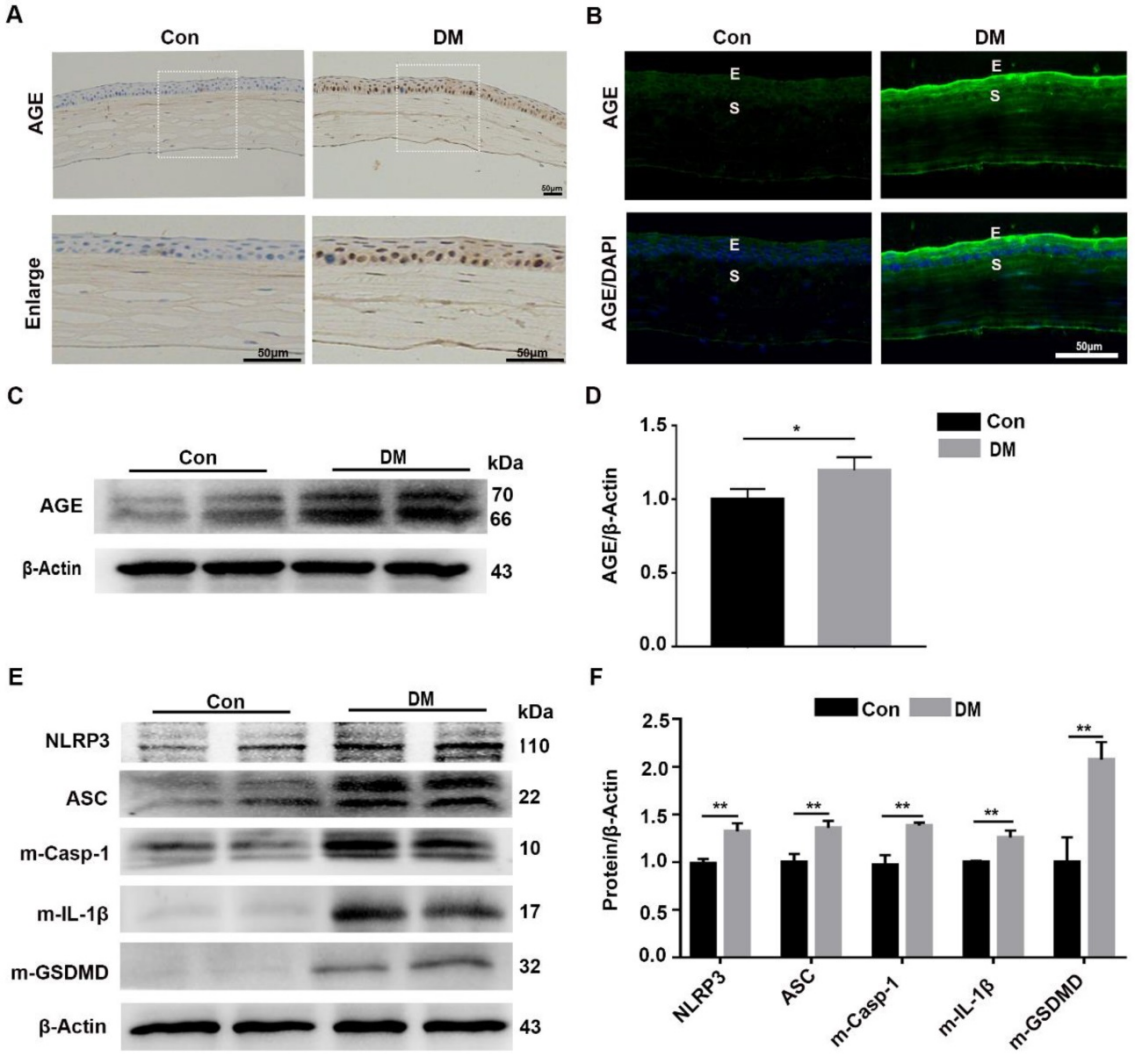
** Diabetic corneas showed increased AGE deposition and basal activation of NLRP3 inflammasome. (A)** The expression and location of AGE in corneas of diabetic and normal mice was examined using immunohistochemistry. **(B)** The level and distribution of AGE in diabetic and normal corneas was evaluated through immunofluorescence staining. E, epithelium; S, stroma. **(C)** The levels of AGEs in diabetic and normal corneas were determined using immunoblotting (n = 3). **(D)** The relative intensities of AGEs in diabetic and normal corneas were quantified as in (C). **(E)** The expression of NLRP3, ASC, m-Casp-1, m-IL-1β and m-GSDMD in diabetic and normal murine corneas was evaluated by western blot. **(F)** The relative levels of NLRP3, ASC, m-Casp-1, m-IL-1β and m-GSDMD in corneas were analysed using Image J software as in (E) (n = 3). The results were presented as mean ±SD. Scale bar, 50 µm. *P <0.05, **P <0.01.

Our results revealed that the sustained NLRP3 inflammasome activation contributed to the impaired diabetic corneal wound healing and nerve regeneration, but the mechanism by which the NLRP3 inflammasome was persistently activated remained uncertain. As an important diabetes-associated endogenous danger-associated molecular pattern (DAMP), AGE products generated by hyperglycemia were reported to be implicated in the pathogenesis of DM and their complications through Toll-like receptors (TLRs) and other receptors 8,29. Therefore, we inferred that the deposited AGE probably contributed to the sustained activation of the NLRP3 inflammasome, subsequently leading to postponed corneal wound healing. The IHC and immunofluorescence staining showed more accumulation of AGEs in mouse diabetic corneas than that in age-matched controls, mainly located at the corneal epithelium, epithelial basement membrane and endothelium (Fig. 4A-B). The western blot analysis further revealed increased accumulation of AGEs in diabetic corneas, when compared with age-matched controls (Fig. 4C-D). Moreover, we also found a higher basal activation of NLRP3 inflammasome in diabetic corneas than in age-matched controls, characterized by elevated matured form of IL-1β and GSDMD, as well as increased expression of NLRP3 and ASC (Fig. 4E-F). Taken together, these results revealed the exacerbated accumulation of AGEs and increased NLRP3 inflammasome activity in diabetic corneas.

### Pharmacologically inhibiting endogenous AGEs formation promoted diabetic corneal epithelial wound healing with reduced NLRP3 inflammasome activation

**Figure 5:**
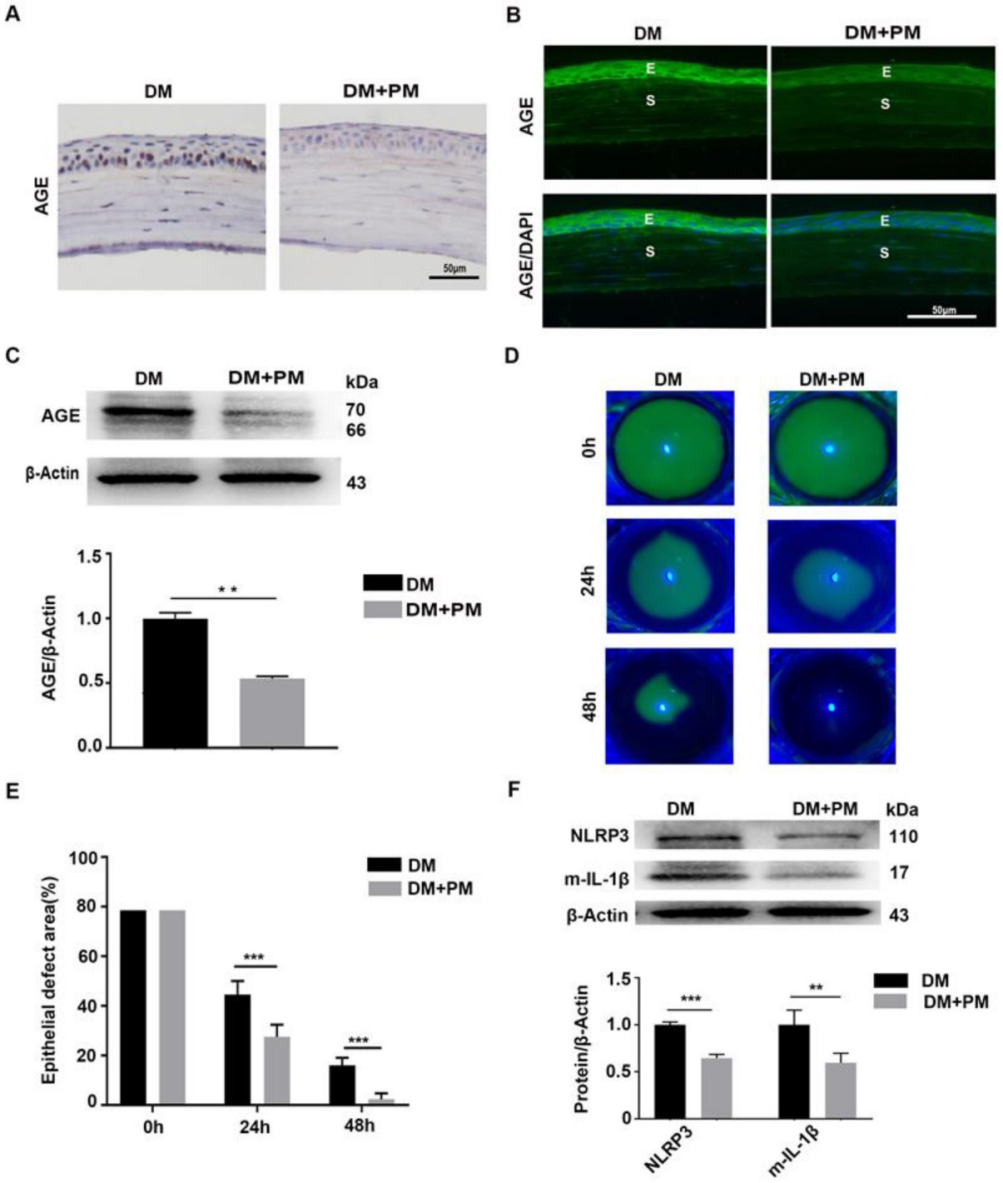
** Pharmacologically inhibiting endogenous AGEs formation accelerated diabetic corneal epithelial wound healing with reduced NLRP3 inflammasome activity. (A)** The content and location of AGEs in diabetic corneas treated with or without PM was determined using immunohistochemistry (n=3). **(B)** The expression and distribution of AGEs in diabetic corneas after treatment with PM was evaluated via immunofluorescence staining (n=3). E, epithelium; S, stroma. **(C)** The levels of AGEs in diabetic corneas treated with or without PM were examined through western blot, and the relative intensities of AGEs (the below) in different groups were quantified by Image J (n=3). **(D)** The representative images of corneal defects in different groups (n=5) at 0 h, 24 h and 48 h after epithelial abrasion were observed by fluorescein sodium staining. **(E)** The relative wound areas of corneas with different groups (n=5) at different time points were calculated by Image J software. **(F)** The protein levels of NLRP3 and m-IL-1β in PM-treated diabetic corneas at 48 hours after epithelial injury were assessed by immunoblotting (n=3). The quantification of NLRP3 and m-IL-1β was performed using Image J. Data were showed as mean ±SD. Scale bar, 50 µm. **P <0.01, ***P <0.01.

To testify whether the deposited AGEs were closely associated with delayed corneal wound healing and increased NLRP3 inflammasome activity in diabetic mice, PM in drinking water was given to diabetic mice for 2 months to inhibit endogenous AGEs formation 24. Through IHC and immunofluorescence staining, reduced AGEs deposition in diabetic corneas was observed after PM treatment than in untreated diabetic corneas (Fig. 5A-B). The western blot analysis also revealed the decreased accumulation of AGEs in diabetic corneas treated with PM (Fig. 5C). These findings indicated that PM treatment significantly blocked AGEs formation. Using corneal abrasion models, we further found that the PM-treated diabetic corneas showed more accelerated epithelial wound closure, when compared with the untreated diabetic mice (Fig. 5D-E). Moreover, the diabetic corneas treated with PM also displayed decreased NLRP3 inflammasome activity, with less production of matured IL-1β and lower expression of NLRP3 (Fig. 5F). These results demonstrated that the accumulated AGEs in diabetic corneas led to the delayed corneal epithelial wound healing probably through activation of the NLRP3 inflammasome.

### Exogenous AGEs promoted NLRP3 inflammasome activation and impaired corneal wound healing and nerve regeneration

**Figure 6:**
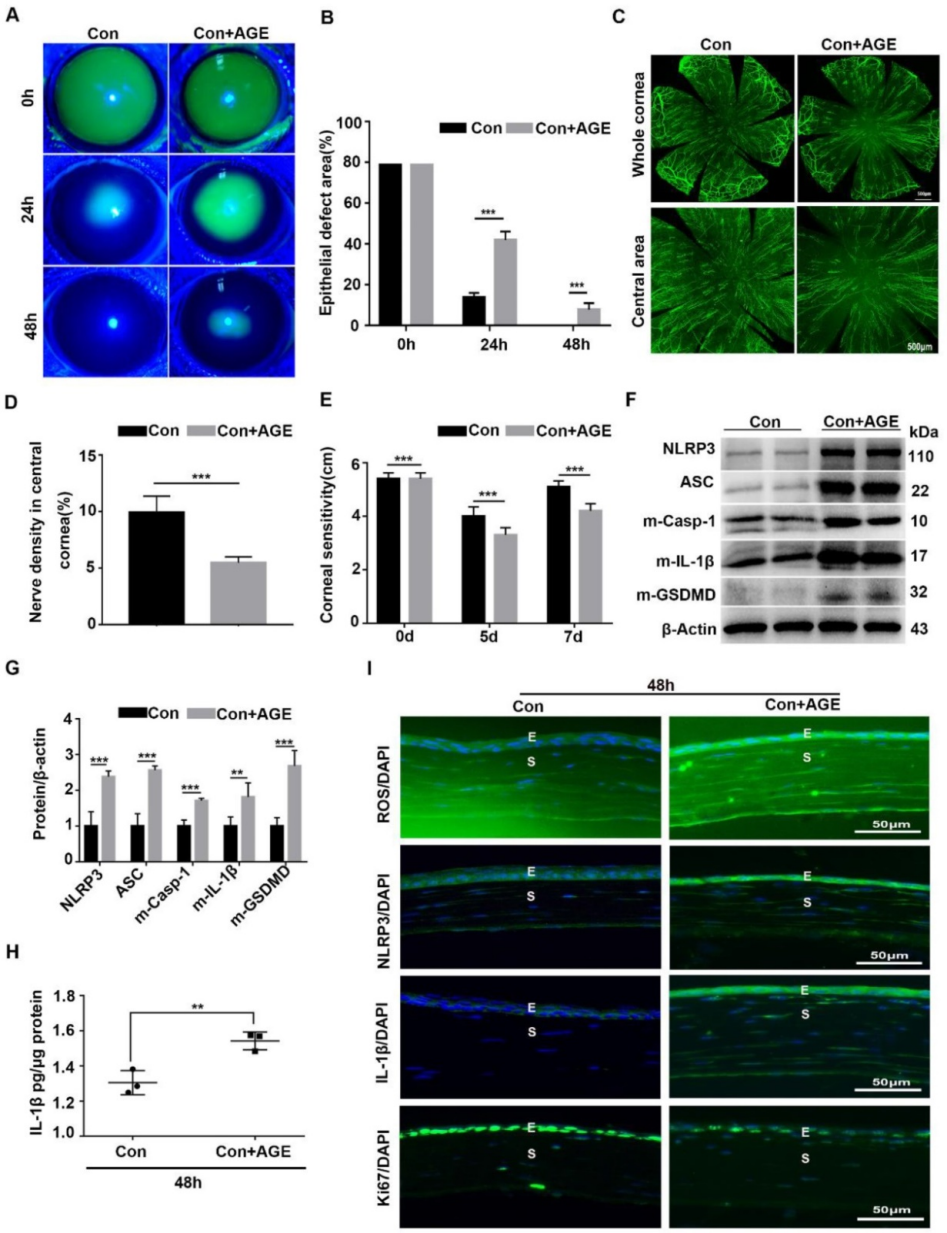
** Exogenous AGEs promoted NLRP3 inflammasome activation and impaired corneal wound healing and nerve regeneration. (A)** The photographs of corneal defects in the two groups at 0 h, 24 h and 48 h after epithelial abrasion were taken by slit-lamp microscope. **(B)** The relative defect area of corneal epithelium with different groups at different time points was quantified using Image J. **(C)** The representative images of the regenerated corneal nerve fibers in different groups were showed by β-tubulin III staining. Above, whole corneas; below, central corneas. **(D)** The quantification of central corneal nerve fibers in different groups (n=5) was performed using Image J. **(E)** The corneal sensitivity at different groups (n=5) detected by Cochet-Bonnet esthesiometer at 5 and 7 d after epithelial abrasion was presented in a histogram. **(F)** The expression of NLRP3, ASC, m-Casp-1, m-IL-1β and m-GSDMD in the corneas in different groups (n=3) was evaluated using western blot. **(G)** The quantification of NLRP3, ASC, m-Casp-1, m-IL-1β and m-GSDMD in corneas of different groups (as in F) was performed using Image J. **(H)** The concentrations of IL-1β in corneas in different groups (n = 3) at 48 h after injury were quantified using ELISA. **(I)** The accumulation of ROS and expression of NLRP3, IL-1β and Ki67 in the cornea were evaluated by immunofluorescence staining. E, epithelium; S, stroma. Data were given as mean ±SD, ** *P* <0.01, *** *P* <0.001.

To further address the effect of AGE on NLRP3 inflammasome activation and corneal wound healing, exogenous AGEs were subconjunctivally injected into normal mice after corneal epithelium scraping. When compared with the untreated mice, AGEs subconjunctival injection significantly delayed corneal epithelial wound healing and impaired nerve regeneration (Fig. 6A-E), with reduced Ki67 positive corneal epithelial cells (Fig. 6I) and decreased STAT3 activity (Supplementary Fig. 4A). Moreover, western blot analysis revealed the increased NLRP3 inflammasome activity in scraped corneas after AGEs treatment, with more production of matured Casp-1, IL-1β and GSDMD and higher expression of NLRP3 and ASC (Fig. 6F-H). The immunofluorescence analysis showed more expression of NLRP3 and IL-1β in scraped corneas after AGEs treatment than in untreated vehicles, further indicative of increased NLRP3 inflammasome activity caused by AGEs (Fig. 6I). Meanwhile, the AGE-treated scraped corneas also showed stronger oxidative stress, as presented by more accumulation of ROS (Fig. 6I) and overexpression of NOX2 and NOX4 (Supplementary Fig. 4B-C). Thus, exogenous AGEs promoted NLRP3 inflammasome activation and oxidative stress, but impaired corneal wound healing and nerve regeneration in normal mice, which were quite similar to the phenotypes obtained in diabetic wound healing.

### AGE-treated TKE2 cells showed increased NLRP3 inflammasome activation but decreased proliferative potential

**Figure 7:**
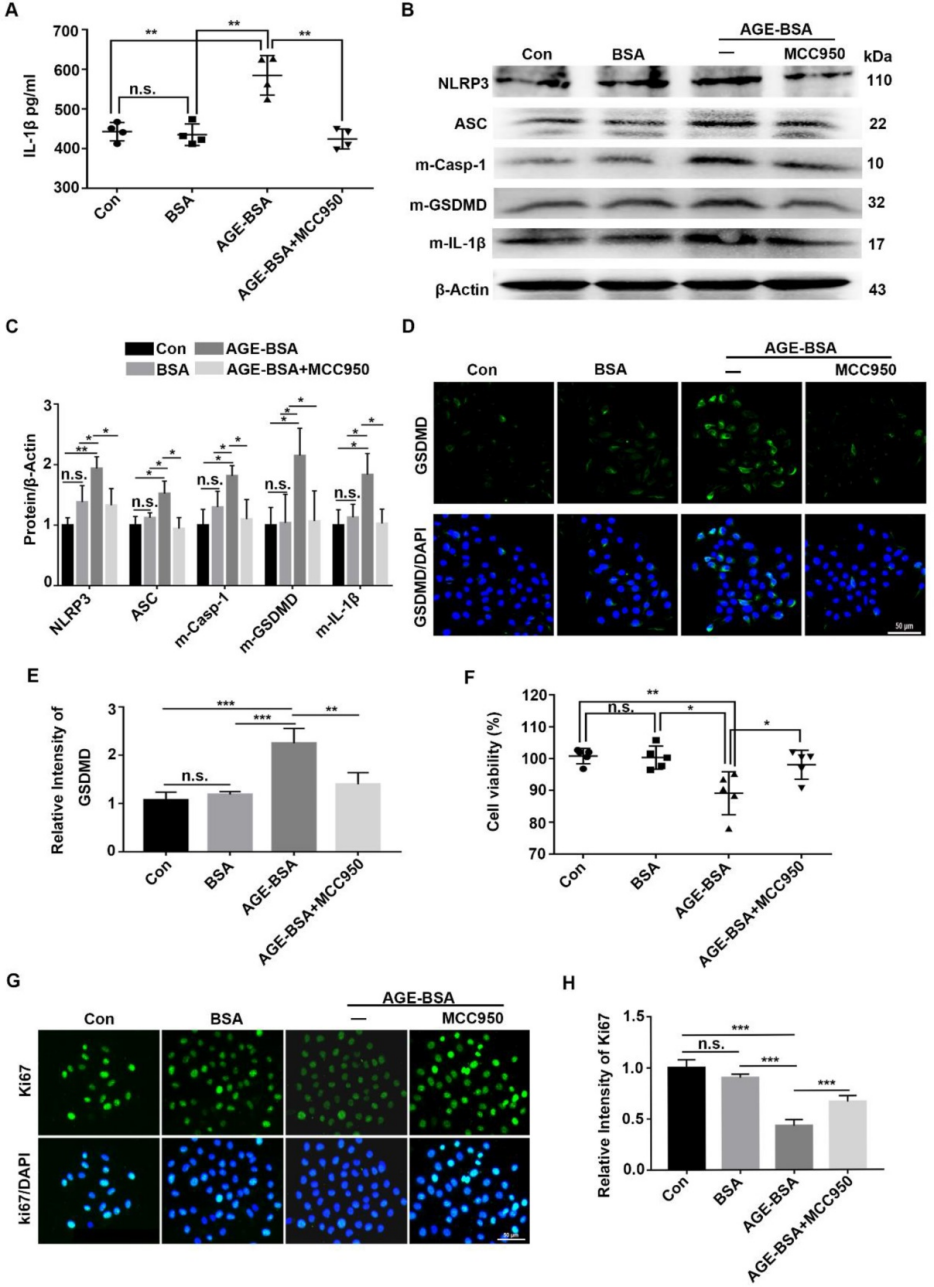
** Exogenous AGEs increased the activation of NLRP3 inflammasome and decreased proliferative potential in TKE2 cells. (A)** The concentrations of IL-1β in supernatants derived from AGE-treated TKE2 in the presence or absence of MCC950 were detected by ELISA (n = 4). **(B)** The protein contents of NLRP3, ASC, m-Casp-1, m-IL-1β and m-GSDMD in TKE2 after treatment with different conditions were examined via immunoblotting. **(C)** The quantification of NLRP3, ASC, m-Casp-1, m-IL-1β and m-GSDMD as in (B) was analyzed by Image J (n=3). **(D)** The expression of GSDMD in TKE2 after treatment with different stimulations was assessed by immunofluorescence staining. **(E)** The relative fluorescence intensity of GSDMD as in (D) was quantified by Image J software. **(F)** The survival of TKE2 after treatment with BSA-AGEs in the presence or absence of MCC950 was evaluated using CCK-8 experiments (n=5). **(G)** The expression of Ki67 in TKE2 cells treated with different stimulations was examined through immunofluorescence staining (n=3). **(H)** The fluorescence intensity of Ki67 as in (G) was quantified by Image J software. The data was reported as mean ±SD. n.s, not significant, * *P* <0.05, ** *P* <0.01, *** *P* <0.001.

To verify the effect of AGEs on NLRP3 inflammasome *in vivo*, the TKE2 cell line was cultured and stimulated with AGE-BSA and MCC950. Through ELISA, we showed higher IL-1β levels in the supernatants of AGE-treated TKE2 than in BSA-treated and normal controls. However, when pre-treated with MCC950, the secretion of IL-1β was significantly inhibited (Fig. 7A). Western blot revealed that AGEs promoted activation of the NLRP3 inflammasome, with a pronounced increase of the matured form of Casp-1(p10), IL-1β and GSDMD, as well as elevated expression of NLRP3 and ASC (Fig. 7B-E). Through immunofluorescence staining, we observed more ASC foci (an indicative of the NLRP3 inflammasome assembly and activation 30) in TKE2 after treatment with AGE-BSA, while MCC950 treatment significantly reversed the BSA-AGE-induced ASC foci formation (Supplementary Fig. 5A). These results further indicated that AGEs contributed to the activation of NLRP3 inflammasome. Moreover, the AGE-treated TKE2 cells also showed aggravated ROS accumulation (Supplementary Fig. 5B), which was in agreement with the findings in AGE-treated mice. In addition to promoting NLRP3 inflammasome activation, AGE-BSA treatment also significantly lowered the proliferative potential and survival rates of TKE2, as presented by decreased cell viability and reduced Ki67 positive cells (Fig. 7F-H). However, MCC950 pre-treatment significantly restored the reduced proliferative potential and survival rates caused by AGE (Fig. 7F-H). Collectively, these results demonstrated that AGEs promoted the activation of the NLRP3 inflammasome, thereby leading to lower proliferative potential and survival rates, probably through inflammation and pyroptosis.

### Scavenging ROS expedited diabetic corneal epithelial wound healing and nerve regeneration through inhibiting NLRP3 inflammasome activation

**Figure 8:**
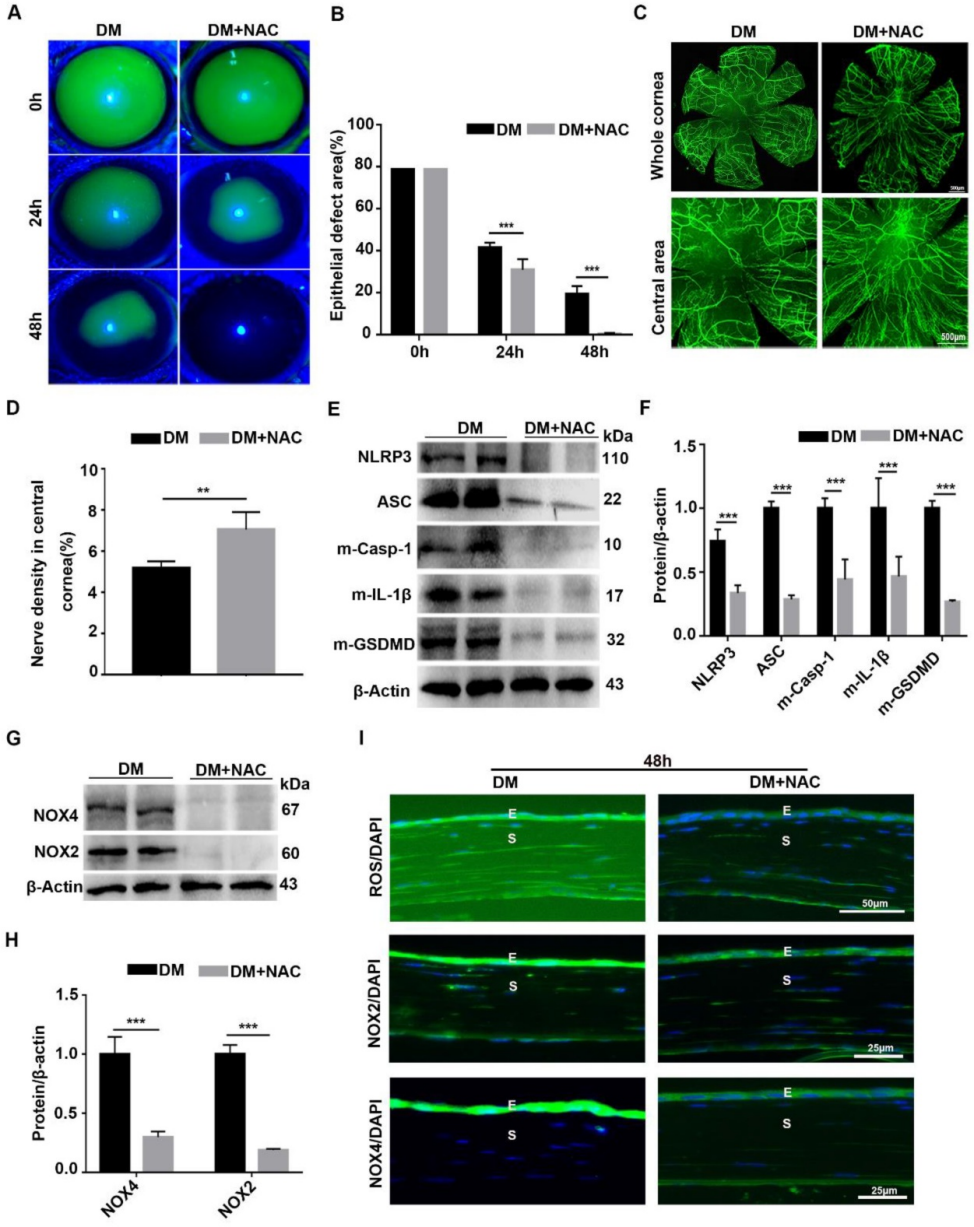
** Scavenging ROS expedited diabetic corneal epithelial wound healing and nerve regeneration by inhibiting NLRP3 inflammasome activation. (A)** Typical photographs of corneal defects in diabetic mice treated or untreated with NAC at different time points were taken by a slit-lamp microscope. **(B)** The relative epithelial defects of corneas in different groups at various time points were analyzed using Image J software (n=5). **(C)** The dynamic changes of regenerated corneal nerve fibers after treatment with NAC at 7days after epithelial injury were examined using β-tubulin III staining. Below, images of central corneas. **(D)** The density of central corneal nerves as in C was quantified by Image J (n=5). **(E)** The protein levels of NLRP3, ASC, m-Casp-1, m-IL-1β and m-GSDMD in the NAC-treated corneas at 48 h after corneal epithelial injury were evaluated by western blot. **(F)** The relative intensity of key proteins as in (E) was quantified (n=3). **(G)** The expression of NOX2 and NOX4 in NAC-treated diabetic corneas was examined by immunoblotting. **(H)** The relative levels of NOX2 and NOX4 as in (G) were determined through Image J software (n=3). **(I)** Immunofluorescence staining was performed to investigate the expression of ROS, NOX2 and NOX4 in NAC-treated diabetic corneas at 48h after abrasion. E, epithelium; S, stroma. Data were presented as mean ±SD, ** *P* <0.01, *** *P* <0.001.

To dissect the association of oxidative stress, NLRP3 inflammasome and diabetic corneal wound healing, the antioxidant NAC was used to evaluate the effect of ROS on the NLRP3 inflammasome and diabetic corneal wound healing. When compared with untreated diabetic mice, the corneal wound closure and nerve regeneration were more dramatically accelerated in NAC-treated diabetic mice (Fig. 8A-D). Consistently, the corneal sensory sensation recovery in treated mice was also significantly improved (Supplementary Fig. 6). These effects were quite similar to the effects achieved by inhibiting NLRP3 inflammasome activity. In addition to the beneficial effect on diabetic corneal wound healing and nerve regeneration, the NAC treated diabetic corneas further displayed lower NLRP3 inflammasome activation than in the untreated vehicles, featured with less production of activated Casp-1(p10), IL-1β and GSDMD, as well as decreased expression of NLRP3 and ASC (Fig. 8E-F). Based on the effective scavenging effect of NAC on oxidative stress (Fig. 8G-H), we concluded that NAC promoted diabetic corneal wound healing and nerve regeneration through blocking the NLRP3 inflammasome.

## Discussion

As the major diabetic complications in the ocular surface, the main clinical manifestations of DK present delayed corneal wound healing and the loss of corneal sensitivity 21,31. However, the underlying pathogenesis of DK is not fully understood, and effective therapeutic approaches remain elusive. In the current study, the main novel finding was that the sustained activation of NLRP3 inflammasome led to postponed diabetic corneal wound healing and impaired nerve regeneration. Mechanistically, the accumulated AGEs contributed to the hyperactivation of NLRP3 inflammasome through ROS generation, ultimately resulting in chronic inflammation and pyroptosis of corneal epithelial cells. Moreover, genetic and pharmacological blockade of the AGEs/ROS/NLRP3 inflammasome axis promoted diabetic corneal wound healing and nerve regeneration.

A growing body of evidence supported NLRP3-inflammasome-mediated inflammation as one of the most important contributors to the pathogenesis of DM and its complications, including diabetes, diabetic nephropathy, diabetic retinopathy, and diabetic cardiomyopathy 6,16,18. Although several mechanisms have been offered to explain the pathogenesis of DK, including disrupted signalling pathways, the loss of trophic support, and increased oxidative stress 31,32, whether NLRP3 inflammasome-mediated chronic inflammation contributed to the development of DK has not been fully investigated. Through genetic and pharmacological approaches, we revealed that the NLRP3 inflammasome was required for corneal wound healing and nerve regeneration under physiological conditions. These results were consistent with other's reports 33-35. However, under diabetic conditions, the hyperactivation of the NLRP3 inflammasome led to delayed corneal wound closure and impaired nerve regeneration, which is in line with the findings from diabetic skin wound healing and diabetic foot ulcer 36-38. These observations supported that chronic low-grade inflammation contributed to the pathogenesis of DK.

Several TLRs were reported to detect the diabetes-associated endogenous DAMPs, including HMGB1 and AGEs, and spark sterile inflammation responses through NF-κB signalling, thereby resulting in diabetic complications 17, 18, 39. Numerous diabetes-associated DAMPs, such as palmitate, lipids, ceramides and amylin, also promote NLRP3 inflammasome activation and lead to pro-inflammatory cascades by induction of IL-1β and IL-18, ultimately contributing to the pathogenesis of diabetes and its complications 7, 40. In this study, we showed more accumulation of AGEs in diabetic corneas than that in normal controls, corresponding with previous reports 41,42. Importantly, the normal mice subconjunctivally treated with AGEs showed delayed corneal wound closure, accompanied with increased NLRP3 inflammasome activity and oxidative stress, as well as decreased expression of Ki67 and p-STAT3 in the corneal epithelium, which was quite similar to DK. However, pharmacologically inhibiting AGEs formation (PM) and ROS scavenger (NAC) treatment significantly expedited diabetic corneal wound healing, along with decreased NLRP3 inflammasome activity, which was in line with the effect of pharmacologically or genetically blocking the NLRP3 inflammasome on diabetic corneal wound healing. Combined with the overexpression of NADPH oxidase (NOX2 and NOX4) during diabetic corneal wound healing, these findings mechanistically demonstrated that the AGE-generated ROS promoted NLRP3 inflammasome activity and inflammatory cascades, resulting in postponed diabetic corneal wound closure and impaired nerve regeneration. As previous studies supported that the ROS derived from NADPH oxidase and mitochondria both contributed to NLRP3 inflammasome activation 27,43-48, further studies are required to determine which kind of ROS promotes AGE-induced NLRP3 inflammasome activation and delayed diabetic corneal wound healing, mitochondria- or NADPH oxidase-generated ROS.

In addition to inflammatory cascades, the activated NLRP3 inflammasome also promoted GSDMD-executed pyroptosis, a kind of programmed cell death 7, 14, 15. Several lines of studies indicated that pyroptosis was involved in the pathogenesis of diabetes and its complications, such as diabetic nephropathy 49, 50, diabetic retinopathy 51-53, and diabetic cardiomyopathy 18, 54, 55. However, whether GSDMD-executed pyroptosis was involved in the pathogenesis of DK remained unknown. During corneal wound closure, we also showed more m-GSDMD formation in diabetic corneas than in control corneas. However, when subconjunctivally treated with MCC950 and antioxidant NAC, the diabetic mice presented accelerated corneal wound healing, with lower m-IL-1β and m-GSDMD. Moreover, the AGE-treated TKE2 cells also exhibited lower proliferative capacity and increased NLRP3 inflammasome activity characterized by higher levels of m-IL-1β and m-GSDMD. These results demonstrated that the diabetic corneal epithelial cells endowing with properties of lower proliferative potential, increased GSDMD-executed pyroptosis and chronic inflammation probably resulted in the postponed corneal wound closure and impaired nerve regeneration. However, further studies are required to investigate the pathological roles of pyroptosis in the pathogenesis of DK.

Wound healing is an important and complicated process for maintaining corneal homeostasis and transparency, involving in various cell types, such as corneal epithelial cells, stem cells, neurons and immune cells 56,57. Although we have demonstrated the different roles of NLRP3 inflammasome in physiological and diabetic corneal wound healing, there are still several limitations in need of resolution. First, it remains uncertain how much epithelial, neural functions are affected by both normal and hyperactivation of NLRP3 inflammasome and how they individually contribute to DK. Second, further study is required to address whether hyperactivated NLRP3 inflammasome is the major driver causing diabetic epithelial or neural dysfunction, which subsequently delayed corneal wound closure. Third, although we determined the critical roles of AGE in activating NLRP3 inflammasome, it is necessary to identify other key diabetes-associated DAMPs contributing to the hyperactivated NLRP3 inflammasome 40.

**Figure 9:**
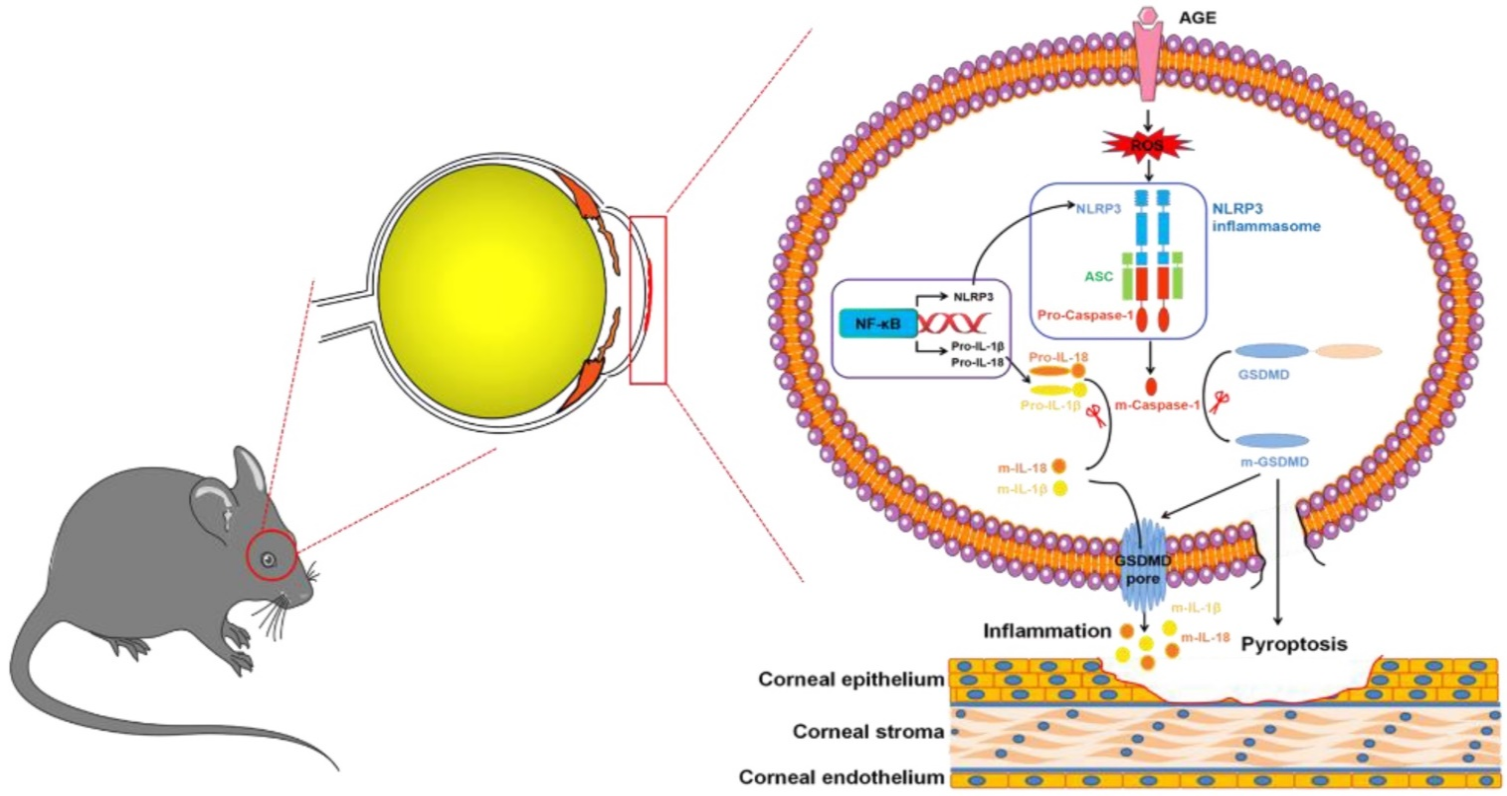
Modes of the AGEs/ROS/NLRP3 inflammasome axis leading to delayed diabetic corneal wound healing.

In summary, we, for the first time, revealed the contributions of NLRP3-inflammasome-mediated chronic inflammation to the pathogenesis of DK. We proposed that the accumulated AGEs promoted NLRP3 inflammasome through ROS, leading to sustained low-grade inflammatory cascades and GSDMD-executed pyroptosis, which ultimately resulted in impaired corneal wound healing and nerve regeneration (Fig. 9). Topically, pharmacological blockade of the NLRP3 inflammasome was sufficient to improve corneal wound healing and nerve regeneration. Our findings not only highlighted the pathogenic roles of the AGEs/ROS/NLRP3 inflammasome axis in the development of DK but also provided a new candidate target for improving diabetic corneal wound healing.

## Supplementary Material

Supplementary figures and table.

## Abbreviations

AGEs: advanced glycation end-products
DK: diabetic keratopathy
STZ: streptozotocin
WT: wild-type
KO: knockout
ROS: reactive oxygen species
GSDMD: gasdermin D
NAC: N-acetyl-cysteine
PM: pyridoxylamine
DM: diabetes mellitus
NLR: NOD-like receptor
ELISA: enzyme-linked immunosorbent assay
IL-1β: interleukin-1β
IL-18: interleukin 18
KSFM: keratinocyte serum-free medium
PBS: phosphate-buffered saline
HKGS: human keratinocyte growth supplement
EGF: epidermal growth factor
RIPA: radioimmunoprecipitation assay
BSA: bovine serum albumin
IHC: immunohistochemistry
NOX2: NADPH oxidase 2
NOX4: NADPH oxidase 4
TLR: Toll-like receptor
STAT3: signal transducer and activator of transcription 3
DAMPs: danger-associated molecular patterns
m-IL-1β: matured IL-1β
m-Casp-1: matured Caspase-1
m-GSDMD: matured GSDMD

## References

[1] Saeedi P, Petersohn I, Salpea P, Malanda B, Karuranga S, Unwin N (2019). Global and regional diabetes prevalence estimates for 2019 and projections for 2030 and 2045: Results from the International Diabetes Federation Diabetes Atlas, 9(th) edition. Diabetes research and clinical practice.

[2] Ljubimov AV, Diabetic complications in the cornea Vis Res. 2017; 139: 138-52.

[3] Calvo-Maroto AM, Perez-Cambrodi RJ, Albaran-Diego C, Pons A, Cervino A (2014). Optical quality of the diabetic eye: a review. Eye (Lond).

[4] Schultz RO, Van Horn DL, Peters MA, Klewin KM, Schutten WH (1981). Diabetic keratopathy. Trans Am Ophthalmol Soc.

[5] Cai D, Zhu M, Petroll WM, Koppaka V, Robertson DM (2014). The impact of type 1 diabetes mellitus on corneal epithelial nerve morphology and the corneal epithelium. Am J Pathol.

[6] Wada J, Makino H (2016). Innate immunity in diabetes and diabetic nephropathy. Nat Rev. Nephrol.

[7] Sharma R, Kanneganti TD (2021). NLRP3 inflammasome in cancer and metabolic diseases. Nat Immunol.

[8] Tang SCW, Yiu WH (2020). Innate immunity in diabetic kidney disease. Nat Rev Nephrol.

[9] Donath MY, Dinarello CA, Mandrup-Poulsen T (2019). Targeting innate immune mediators in type 1 and type 2 diabetes. Nat Rev Immunol.

[10] Wen H, Ting JP, O'Neill LA (2012). A role for the NLRP3 inflammasome in metabolic diseases-did Warburg miss inflammation?. Nat Immunol.

[11] Swanson KV, Deng M, Ting JP (2019). The NLRP3 inflammasome: molecular activation and regulation to therapeutics. Nat Rev Immunol.

[12] Christgen S, Place DE, Kanneganti TD (2020). Toward targeting inflammasomes: insights into their regulation and activation. Cell Res.

[13] Christgen S, Kanneganti TD (2020). Inflammasomes and the fine line between defense and disease. Curr Opin Immunol.

[14] Shi J, Zhao Y, Wang K, Shi X, Wang Y, Huang H (2015). Cleavage of GSDMD by inflammatory caspases determines pyroptotic cell death. Nature.

[15] Kayagaki N, Stowe IB, Lee BL, O'Rourke K, Anderson K, Warming S (2015). Caspase-11 cleaves gasdermin D for non-canonical inflammasome signalling. Nature.

[16] Thounaojam MC, Montemari A, Powell FL, Malla P, Gutsaeva DR, Bachettoni A (2019). Monosodium Urate Contributes to Retinal Inflammation and Progression of Diabetic Retinopathy. Diabetes.

[17] Chaurasia SS, Lim RR, Parikh BH, Wey YS, Tun BB, Wong T Y (2018). The NLRP3 Inflammasome May Contribute to Pathologic Neovascularization in the Advanced Stages of Diabetic Retinopathy. Sci Rep.

[18] Xie Y, Huang Y, Ling X, Qin H, Wang M, Luo B (2020). Chemerin/CMKLR1 Axis Promotes Inflammation and Pyroptosis by Activating NLRP3 Inflammasome in Diabetic Cardiomyopathy Rat. Front Physiol.

[19] Sharma A, Choi JSY, Stefanovic N, Al-Sharea A, Simpson D S, Mukhamedova N (2021). Specific NLRP3 Inhibition Protects Against Diabetes-Associated Atherosclerosis. Diabetes.

[20] Huang W, Jiao J, Liu J, Huang M, Hu Y, Ran W (2020). MFG-E8 accelerates wound healing in diabetes by regulating "NLRP3 inflammasome-neutrophil extracellular traps" axis. Cell Death Discov.

[21] Qing L, Fu J, Wu P, Zhou Z, Yu F, Tang J (2019). Metformin induces the M2 macrophage polarization to accelerate the wound healing via regulating AMPK/mTOR/NLRP3 inflammasome singling pathway. Am J Transl Res.

[22] Di G, Zhao X, Qi X, Zhang S, Feng L, Shi W (2017). VEGF-B promotes recovery of corneal innervations and trophic functions in diabetic mice. Sci Rep.

[23] Dong M, Di G, Zhang X, Zhou Q, Shi W (2017). Subconjunctival Bevacizumab Injection Impairs Corneal Innervations and Epithelial Wound Healing in Mice. Invest Ophthamol Vis Sci.

[24] Stitt A, Gardiner TA, Alderson NL, Canning P, Frizzell N, Duffy N The AGE inhibitor pyridoxamine inhibits development of retinopathy in experimental diabetes Diabetes. 2002; 51(9): 2826-32.

[25] Wang X, Li W, Zhou Q, Li J, Zhang J, Li D (2020). MANF Promotes Diabetic Corneal Epithelial Wound Healing and Nerve Regeneration by Attenuating Hyperglycemia-Induced Endoplasmic Reticulum Stress. Diabetes.

[26] Kawakita T, Shimmura S, Hornia A, Higa K, Tseng S C (2008). Stratified epithelial sheets engineered from a single adult murine corneal/limbal progenitor cell. J Cell Mol Med.

[27] Chen Z, Wu C, Liu Y, Li H, Zhu Y, Huang C (2020). ELABELA attenuates deoxycorticosterone acetate/salt-induced hypertension and renal injury by inhibition of NADPH oxidase/ROS/NLRP3 inflammasome pathway. Cell Death Dis.

[28] Wang X, Zhang S, Dong M, Li Y, Zhou Q, Yang L (2020). The proinflammatory cytokines IL-1β and TNF-α modulate corneal epithelial wound healing through p16Ink4a suppressing STAT3 activity. J Cell Physiol.

[29] Wang Y, Luo W, Han J, Khan ZA, Fang Q, Jin Y (2020). MD2 activation by direct AGE interaction drives inflammatory diabetic cardiomyopathy. Nat Commun.

[30] Song N, Li T (2018). Regulation of NLRP3 Inflammasome by Phosphorylation. Front Immunol.

[31] Kim J, Kim CS, Sohn E, Jeong IH, Kim H, Kim J.S (2011). Involvement of advanced glycation end products, oxidative stress and nuclear factor-kappaB in the development of diabetic keratopathy. Graefes Arch Clin Exp Ophthalmol.

[32] Zhu L, Titone R, Robertson DM (2019). The impact of hyperglycemia on the corneal epithelium: Molecular mechanisms and insight. The Ocular Surface.

[33] Ito H, Kanbe A, Sakai H, Seishima M (2018). Activation of NLRP3 signalling accelerates skin wound healing. Exp Dermatol.

[34] Vinaik R, Abdullahi A, Barayan D, Jeschke MG (2020). NLRP3 inflammasome activity is required for wound healing after burns. Transl Res.

[35] Weinheimer-Haus EM, Mirza RE, Koh TJ (2015). Nod-like receptor protein-3 inflammasome plays an important role during early stages of wound healing. PloS One.

[36] Wong SL, Demers M, Martinod K, Gallant M, Wang Y, Goldfine AB (2015). Diabetes primes neutrophils to undergo NETosis, which impairs wound healing. Nat Med.

[37] Fadini GP, Menegazzo L, Rigato M, Scattolini V, Poncina N, Bruttocao A (2016). NETosis Delays Diabetic Wound Healing in Mice and Humans. Diabetes.

[38] Liu C, Teo MHY, Pek SLT, Wu X, Leong M L, Tay H M (2020). A Multifunctional Role of Leucine-Rich alpha-2-Glycoprotein 1 in Cutaneous Wound Healing Under Normal and Diabetic Conditions. Diabetes.

[39] Gong T, Liu L, Jiang W, Zhou R (2020). DAMP-sensing receptors in sterile inflammation and inflammatory diseases. Nat Rev Immunol.

[40] Shin JJ, Lee EK, Park TJ, Kim W (2015). Damage-associated molecular patterns and their pathological relevance in diabetes mellitus. Ageing Res Rev.

[41] Kaji Y, Usui T, Oshika T, Matsubara M, Yamashita H, Araie M (2000). Advanced glycation end products in diabetic corneas. Investig Ophthamol Vis Sci.

[42] Zou C, Wang S, Huang F, Zhang Y A (2012). Advanced glycation end products and ultrastructural changes in corneas of long-term streptozotocin-induced diabetic monkeys. Cornea.

[43] Han Y, Xu X, Tang C, Gao P, Chen X, Xiong X (2018). Reactive oxygen species promote tubular injury in diabetic nephropathy: The role of the mitochondrial ros-txnip-nlrp3 biological axis. Redox Biol.

[44] Hoyt LR, Randall MJ, Ather JL, DePuccio DP, Landry CC, Qian X (2017). Mitochondrial ROS induced by chronic ethanol exposure promote hyper-activation of the NLRP3 inflammasome. Redox Biol.

[45] Wynosky-Dolfi MA, Snyder AG, Philip NH, Doonan PJ, Poffenberger MC, Avizonis D (2014). Oxidative metabolism enables Salmonella evasion of the NLRP3 inflammasome. J Exp Med.

[46] Tschopp J, Schroder K (2010). NLRP3 inflammasome activation: The convergence of multiple signalling pathways on ROS production?. Nat Rev Immunol.

[47] Zhao Y, Wang Z, Feng D, Zhao H, Lin M, Hu Y (2019). p66Shc Contributes to Liver Fibrosis through the Regulation of Mitochondrial Reactive Oxygen Species. Theranostics.

[48] Zhao Y, Qiu C, Wang W, Peng J, Cheng X, Yang T (2020). Cortistatin protects against intervertebral disc degeneration through targeting mitochondrial ROS-dependent NLRP3 inflammasome activation. Theranostics.

[49] Xie C, Wu W, Tang A, Luo N, Tan Y (2019). lncRNA GAS5/miR-452-5p Reduces Oxidative Stress and Pyroptosis of High-Glucose-Stimulated Renal Tubular Cells. Diabetes Metab Syndr Obes.

[50] Liu P, Zhang Z, Li Y (2021). Relevance of the Pyroptosis-Related Inflammasome Pathway in the Pathogenesis of Diabetic Kidney Disease. Front Immunol.

[51] Gu C, Draga D, Zhou C, Su T, Zou C, Gu Q (2019). miR-590-3p Inhibits Pyroptosis in Diabetic Retinopathy by Targeting NLRP1 and Inactivating the NOX4 Signaling Pathway. Investig Pphthalmol Vis Sci.

[52] Yang K, Liu J, Zhang X, Ren Z, Gao L, Wang Y (2020). H3 Relaxin Alleviates Migration, Apoptosis and Pyroptosis Through P2X7R-Mediated Nucleotide Binding Oligomerization Domain-Like Receptor Protein 3 Inflammasome Activation in Retinopathy Induced by Hyperglycemia. Front Pharmacol.

[53] Yumnamcha T, Devi TS, Singh LP (2019). Auranofin Mediates Mitochondrial Dysregulation and Inflammatory Cell Death in Human Retinal Pigment Epithelial Cells: Implications of Retinal Neurodegenerative Diseases. Front Neurosci.

[54] Xu Y, Fang H, Xu Q, Xu C, Yang L, Huang C (2020). LncRNA GAS5 inhibits NLRP3 inflammasome activation-mediated pyroptosis in diabetic cardiomyopathy by targeting miR-34b-3p/AHR. Cell Cycle.

[55] Yang F, Qin Y, Wang Y, Meng S, Xian H, Che H (2019). Metformin Inhibits the NLRP3 Inflammasome via AMPK/mTOR-dependent Effects in Diabetic Cardiomyopathy. Int J Biol Sci.

[56] Puri S, Sun M, Mutoji KN, Gesteira TF, Coulson-Thomas VJ (2020). Epithelial Cell Migration and Proliferation Patterns during Initial Wound Closure in Normal Mice and an Experimental Model of Limbal Stem Cell Deficiency. Invest Ophthalmol Vis Sci.

[57] Yan D, Yu F, Chen L, Yao Q, Yan C, Zhang S (2020). Subconjunctival Injection of Regulatory T Cells Potentiates Corneal Healing Via Orchestrating Inflammation and Tissue Repair After Acute Alkali Burn. Invest Ophthalmol Vis Sci.

