# Supplementary material for: The advanced glycation end-products (AGEs)/ROS/NLRP3 inflammasome axis contributes to delayed diabetic corneal wound healing and nerve regeneration
Source: Int J Biol Sci. 2022 Jan 1;18(2):809–25. doi: 10.7150/ijbs.63219 (PMC8741862; doi:10.7150/ijbs.63219)
Supplement: Supplementary file 1 — Supplementary figures and table. [file ijbsv18p0809s1.pdf]

Suppl.Table.1 Antibodies for western blotting, immunofluorescence and immunohistochemistry

Primary antibody	Dilution concentration	Supplier	Code
Anti-ANLRP3	WB (1/1000) IF (1/200) IHC (1/200)	Cell Signaling Technology	15101
Anti- ASC	WB (1/1000)	Cell Signaling Technology	67824
Anti- Caspase-1	WB (1/1000)	Cell Signaling Technology	24232
Anti-IL-1β	WB (1/1000) IHC (1/200)	Cell Signaling Technology	31202
Anti-IL-1β	IF (1/200)	Abcam	ab9722
Anti- GSDMD	WB (1/1000) IF (1/200)	Abcam	ab209845
Anti-AGE	WB (1/1000) IF (1/200) IHC (1/100)	BIOSS	Bs-1158R
Anti-NADPH oxidase 2	WB (1/1000) IF (1/200)	Abcam	ab129068
Anti-NADPH oxidase 4	WB (1/1000) IF (1/200)	Abcam	ab133303
Anti-Ki67	IF (1/200)	Abcam	ab16667
Anti-β-actin	WB (1/2000)	Cell Signaling Technology	4970
Anti-p-Stat3	IF (1/200)	Abcam	ab68153
anti -Tubulin Beta 3（TUBB3）	IF (1/200)	Biolegend	657403
Alexa Fluor 488 goat anti-rabbit IgG	IF (1/200)	Beyotime	A0423
Goat anti-rat IgG	WB (1/3000)	Proteintech	SA00001-2

Abbreviations: WB, western blotting; IF, immunofluorescence; IHC, immunohistochemistry

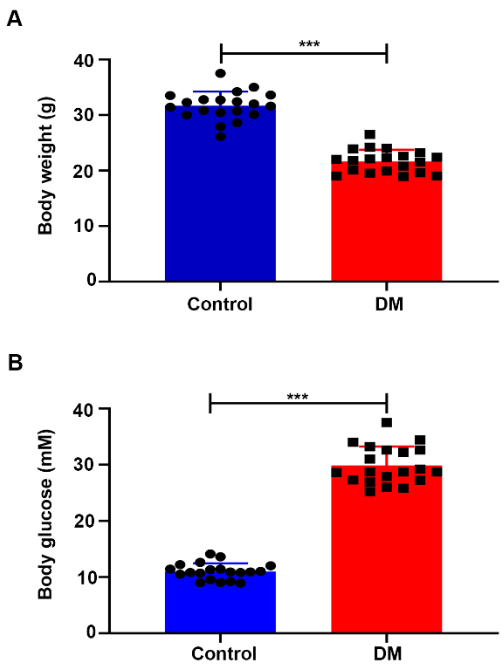

Suppl. Fig. 1 Body weight and glucose of normal mice and type 1 diabetic mice. (A) Body weight of normal and type 1 diabetic mice. (B) Blood glucose of normal and type 1 diabetic mice. (n = 20/ group). ***P < 0.001.

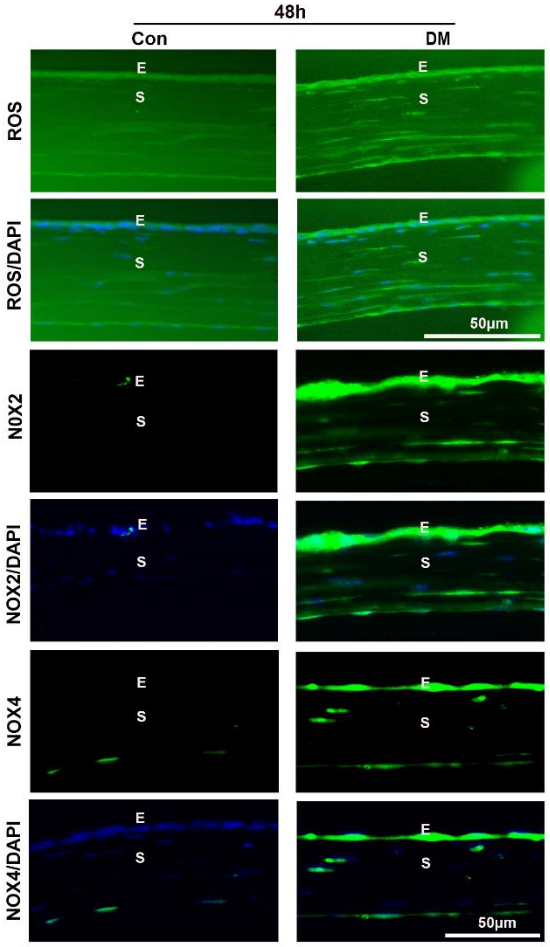

Suppl. Fig. 2. The diabetic corneas displayed increased levels of ROS, NOX2 and NOX4 at 48h after corneal epithelial abrasion. The levels of ROS, NOX2 and NOX4 in corneas at 48h after corneal epithelial abrasion were detected using immunofluorescence staining. E, epithelium; S, stroma.

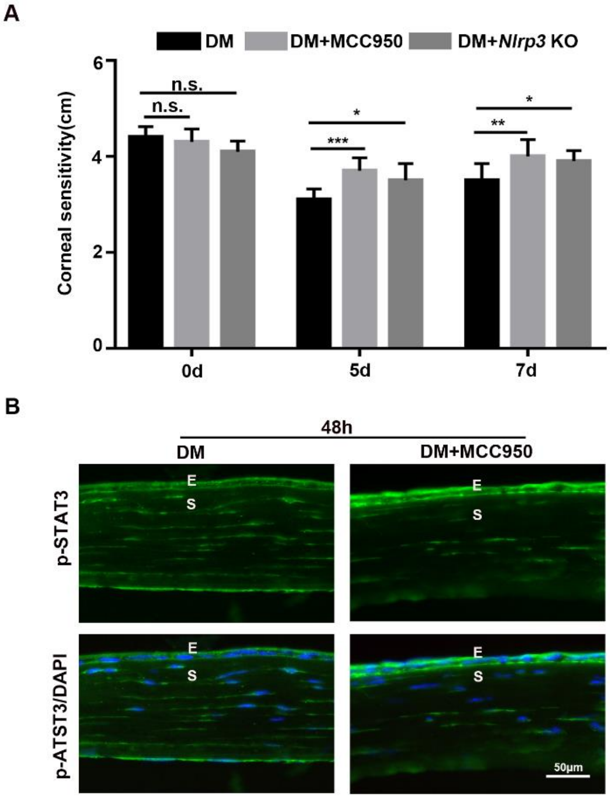

Suppl. Fig. 3. Blocking NLRP3 inflammasome activation accelerated the corneal sensitivity recovery and increased signal transducer and activator of transcription 3 (STAT3) activity in diabetic mice. (A) The corneal sensation at 5 and 7 d after corneal epithelial removal was tested by a Cochet-Bonnet esthesiometer (n=5). (B) The expression of p-STAT3 in the corneas at 48 h after epithelial injury was determined using immunofluorescence staining. E, epithelium; S, stroma. Data were showed as mean ±SD. n.s, not significant, * P <0.05, ** P <0.01, *** P <0.001.

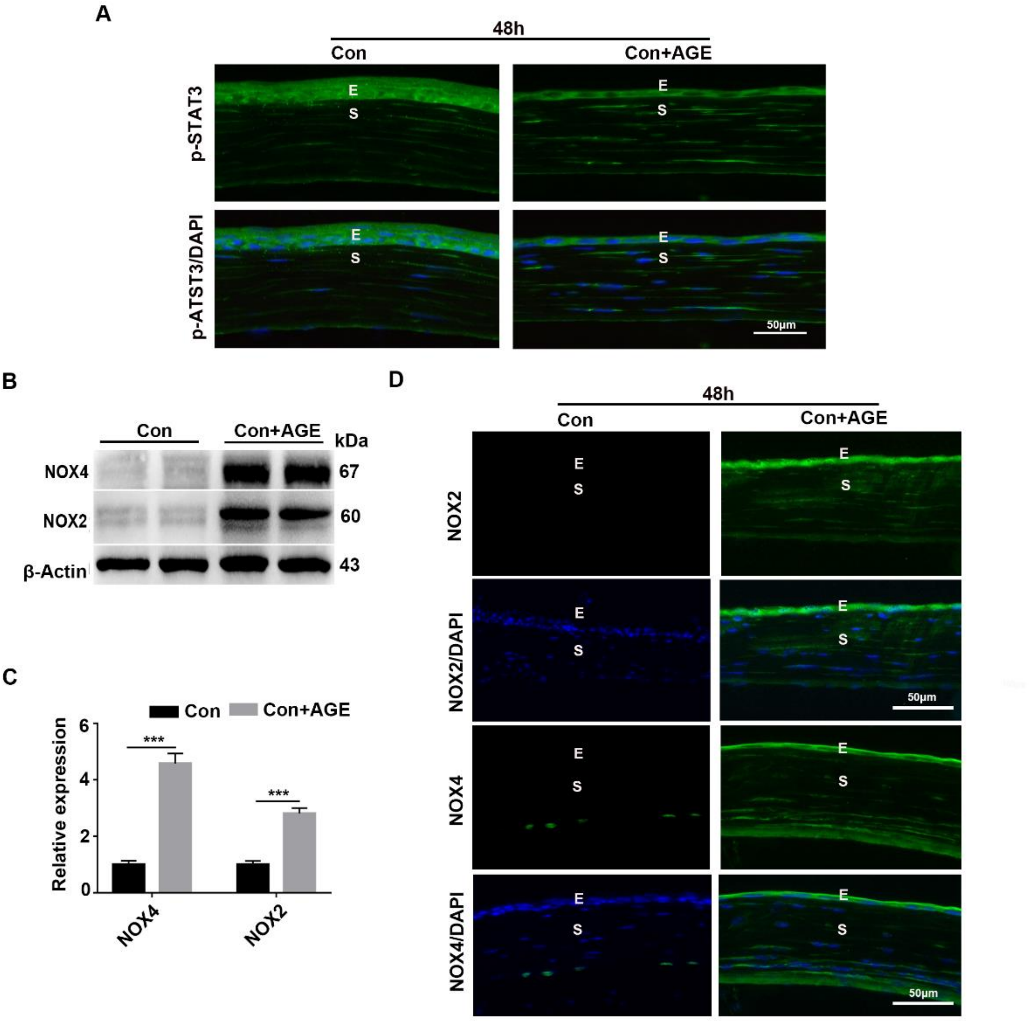

Suppl. Fig. 4. Exogenous AGEs promoted oxidative stress and inhibited STAT3 activation in normal mice. (A) The activation of STAT3 in the cornea at 48 h after epithelial injury was determined by p-STAT3 staining (n=3). (B) The levels of NOX2 and NOX4 in AGE-treated corneas at 48h after epithelial abrasion were tested by western blot. (C) The relative expression of NOX2 and NOX4 as in (B) was quantified by Image J (n=3). (D) The expression of NOX2 and NOX4 in the corneas at 48 h after epithelial injury was examined using immunofluorescence staining (n=3). Data were presented as mean ±SD. E, epithelium; S, stroma. Data were showed as mean ±SD. *** P <0.001.

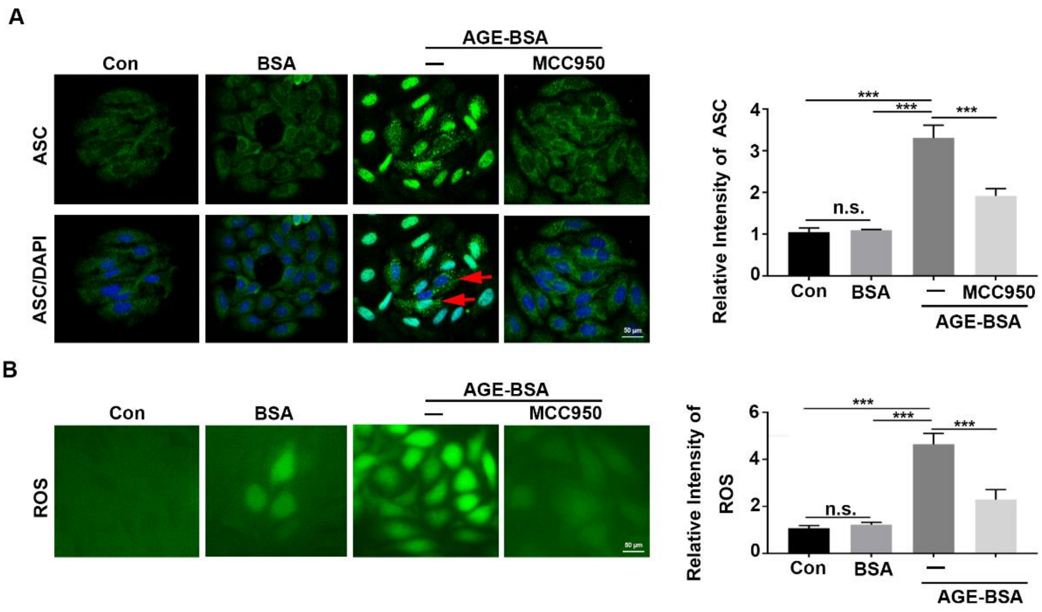

Suppl. Fig. 5. AGEs promoted NLRP3 inflammasome activation and accumulation of ROS in TKE2 cells. (A) The ASC in AGE-BSA-treated TKE2 in the presence or absence of MCC950 was evaluated by immunofluorescence staining (n=3). Left, the intensity of ASC in different groups was quantified by Image J software. The ASC foci were shown by red arrows. (B) The ROS accumulation in AGE-BSA-treated TKE2 in the presence or absence of MCC950 was evaluated by DCFH-DA. Left, the intensity of ROS in different groups was determined by Image J software. Data were showed as mean ±SD. n.s, not significant, *** P <0.001.

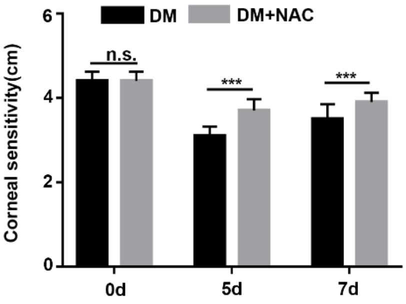

Suppl. Fig. 6. NAC accelerated the corneal sensitivity recovery in diabetic mice. The corneal sensation was tested by a Cochet-Bonnet esthesiometer at 5 and 7 d after epithelial removal in the diabetic mice treated or untreated with NAC (n=5). Data were given as mean ±SD, n.s, not significant, *** P <0.001.
